# Ghrelin rescues skeletal muscle catabolic profile in the R6/2 mouse model of Huntington’s disease

**DOI:** 10.1038/s41598-017-13713-5

**Published:** 2017-10-24

**Authors:** Marie Sjögren, Ana I. Duarte, Andrew C. McCourt, Liliya Shcherbina, Nils Wierup, Maria Björkqvist

**Affiliations:** 1 Wallenberg Neuroscience Center, Department of Experimental Medical Sciences, Brain Disease Biomarker Unit, Lund University, Lund, Sweden; 2 CNC - Center for Neuroscience and Cell Biology, Rua Larga, Faculty of Medicine (Pólo 1, 1st Floor), University of Coimbra, 3004-517 Coimbra, Portugal; 3 Institute for Interdisciplinary Research (IIIUC), University of Coimbra, Casa Costa Alemão - Pólo II, Rua D. Francisco de Lemos, 3030-789 Coimbra, Portugal; 4 Lund University Diabetes Centre, Neuroendocrine Cell Biology, Department of Clinical Sciences in Malmö, Clinical research center, Lund University, Malmö, Sweden

## Abstract

Accumulating evidence suggests altered energy metabolism as a key feature in Huntington’s disease (HD) pathology. Hyper-catabolism, including weight loss and muscle atrophy, is seen in HD patients and HD mouse models. Metabolic hormones are key players, not only in energy metabolism, but also in neurodegenerative processes. Ghrelin, a gut peptide-hormone, plays an important role in regulating energy metabolism, stimulating appetite, and affects brain function and increases neuronal survival. The R6/2 mouse model of HD has previously been shown to exhibit progressive weight loss, dysregulated glucose metabolism, skeletal muscle atrophy and altered body composition. In this study, we targeted energy metabolism in R6/2 mice using ghrelin administration, with the primary aim to delay weight loss and reduce muscle atrophy. We also evaluated glucose metabolism and behaviour. We here demonstrate that ghrelin administration (subcutaneous 150 μg/kg daily injections) for 4 weeks, reversed the catabolic gene expression profile (increased expression of *Caspase 8*, *Traf-5* and *Creb1*) seen in R6/2 mouse skeletal muscle. Skeletal muscle morphology was also improved with ghrelin, and importantly, ghrelin administration normalized behavioural deficits in R6/2 mice. Taken together, our findings encourage further studies targeting metabolism in HD.

## Introduction

The inherited neurodegenerative disorder Huntington’s disease (HD) has over the last decade emerged as a whole body disorder. HD is caused by a CAG triplet repeat expansion in the gene encoding huntingtin^1^. Although HD pathology is traditionally considered to arise from degeneration of basal ganglia and cortex, the ubiquitous expression of the causative mutant huntingtin protein^2^ results in peripheral pathology evident from the early stages of the disease in both human HD and HD mice. Weight loss, altered energy metabolism and systemic low-grade immune response are accompanied by severe muscle wasting, altered distribution of adipose tissue and endocrine disturbances^3–5^. Weight loss and muscle wasting are comorbidities associated with a wide range of disorders and severely affect patient prognosis and quality of life. Muscle wasting is a well-recognized phenomenon in patients with HD that worsens with disease progression^6,7^ and is replicated in some HD mouse models^8^.

Somatotrophic hormones, such as growth hormone (GH), growth hormone releasing hormone (GHRH) and growth hormone secretagogues play important roles in whole body energy metabolism, as well as in brain function^9^. Somatotrophic hormones exert effects, not only on bone growth and skeletal muscle anabolism, but also on body fat distribution (reviewed in ref.^10^).

In human HD, as well as in mouse models of HD, endocrine alterations are present throughout the disease course^11,12^. Changes in the regulation of GH secretion dynamics have been described in early stage HD patients^13^ and dietary anaplerotic therapy has shown beneficial effects on energy metabolism in HD subjects^14^. Ghrelin, a peptide hormone produced by endocrine cells, P/D1 cells, in the gastrointestinal tract^15,16^, has wide-spread multi-tissue effects through signalling pathways involving the GH secretagogue receptor 1a (GHS-R1a)^17^. Ghrelin has effects on tissues other than the pituitary and hypothalamus, such as skeletal muscle, modulating skeletal muscle mass, pancreatic islets and adipose tissue, modulating glucose and lipid metabolism, and in immune cells, modulating inflammatory response (reviewed in ref.^17^).

GHS-R1a agonists have been shown to have beneficial effects for many clinical problems, including muscle wasting, cancer cachexia, cognitive decline, diabetes and metabolic disorders^18–20^. In addition, ghrelin analogues exert beneficial effects on brain function and cognition, increasing neuronal survival by reducing apoptosis^21^.

In the present study, we therefore investigated the effect of ghrelin on weight loss, muscle atrophy and features of metabolism in the R6/2 mouse model of HD. In addition, we evaluated possible effects of ghrelin administration on behaviour.

## Results

### Ghrelin moderately postpones weight loss in R6/2 mice

**Figure 1:**
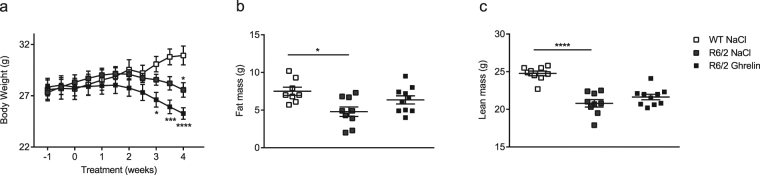
Ghrelin administration moderately affects body weight loss in R6/2 mice. Body weight was assessed twice weekly for 5 weeks, starting one week prior to ghrelin or vehicle administration. Significant body weight loss was seen in vehicle treated R6/2 mice after 3 weeks of treatment compared to WT littermates. Ghrelin administration postponed R6/2 mouse weight loss by one week (**a**). Body composition was assessed using DexaScan, in R6/2 mice and WT littermates after 4 weeks of ghrelin or vehicle administration. There was a significant decrease in fat (**b**) and lean mass (**c**) in R6/2 mice compared to WT mice, but Ghrelin administration did not significantly alter body composition. Data represent mean ± SEM, N = 9–11/group. Statistical significance was determined by 2-way ANOVA with Bonferroni post hoc test for multiple comparisons. *p < 0.05, ****p < 0.0001.

The R6/2 mouse model mimics human HD and displays progressive weight loss^11,22^. Body weight was monitored twice weekly for 5 weeks, commencing at 9 weeks of age, one week prior to daily ghrelin administration (s.c), that started at 10 weeks of age. We here confirm the expected progressive weight loss in R6/2 mice (Fig. 1). Following the first week of treatment, vehicle-treated R6/2 mice lacked weight gain compared with wild type (WT) mice, whereas ghrelin-treated R6/2 mice had a body weight curve that did not differ from vehicle-treated WT littermates (Fig. 1). After 3 weeks of ghrelin-treatment, a significantly lower body weight was observed in R6/2 mice treated with vehicle compared with vehicle-treated WT mice. Weight loss was postponed by one week in ghrelin-treated R6/2 mice (Fig. 1). At the end of study, at 14 weeks of age, mean weight was 30.92 g for vehicle treated WT mice (30.92 ± 2.02), 25.27 g for vehicle treated R6/2 mice (25.27 ± 1.32) and 27.57 g for ghrelin-treated R6/2 mice (27.57 ± 1.97).

Wild-type mice gained weight in response to ghrelin administration (Supplementary Fig. 1) in agreement with previous studies^23,24^.

[weight; genotype + treatment p = 0,0001, F(3, 36) = 9, 295; time points p < 0,0001, F(10, 360) = 13; interaction p < 0,0001, F(30, 360) = 11, 05; subjects (matching) p < 0,0001, F(36, 360) = 36, 78].

### Ghrelin does not significantly alter body composition in R6/2 mice

As previously shown^25^, we could here confirm that body fat mass (g) and body lean mass (g) are significantly reduced in 14-week old R6/2 mice compared with WT littermates (*p* < 0.05, *p* < 0.0001 respectively) (Fig. 1). There was no significantly altered body fat or body lean mass in either WT (Supplementary Fig. 2a and b) or R6/2 mice (Fig. 1a and b) in response to ghrelin administration. Body composition after 2 weeks of ghrelin treatment was not altered (Supplementary Fig. 2c and d). [Fat mass; genotype p = 0.0031, F(1, 34) = 10.16; treatment p < 0.0001, F(1, 34) = 28.31; interaction p = 0.8055, F(1, 34) = 0.06158. Lean mass; genotype p = 0.0059, F(1, 35) = 8.611; treatment p < 0.0001, F(1, 35) = 113.7; interaction p = 0.3800, F(1, 35) = 0.7904].

### Ghrelin reverses R6/2 mouse skeletal muscle atrophic features

**Figure 2:**
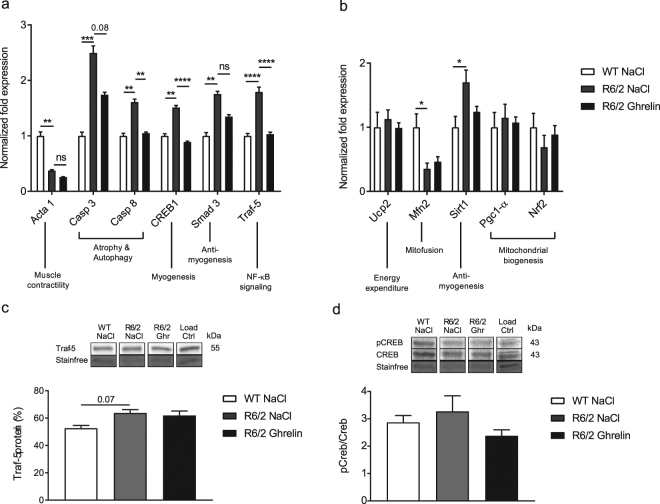
Ghrelin administration (for 4 weeks) normalizes altered gene expression profile in R6/2 mouse skeletal muscle. Normalized skeletal muscle (gastrocnemius) gene expression related to muscle contractility and atrophy (**a**) and mitochondrial biogenesis (**b**) at 14 weeks and after 4 weeks of ghrelin administration. Expression of *Acta1* was significantly decreased, while the expression of *Casp3*, *Casp8*, *Creb1*, *Smad3* and *Traf-5* were significantly increased in R6/2 compared to WT mice. 4 weeks of ghrelin administration significantly normalized *Casp8*, *Creb1 and Traf-5* expression (**a**). Normalized mitochondrial gene expression at 14 weeks illustrates significantly decreased expression of *Mfn2*, while the expression of *Sirt1* was significantly increased. There was no significant effect of ghrelin administration (**b**). In panel c and d representative Western blot and densitometry of protein levels at 14 weeks are shown (**c**,**d**). We could see a trend for upregulation of TRAF-5 protein intensity (**c**) and activated CREB levels (**d**) in vehicle treated R6/2 mice compared to WT littermates. Ghrelin administration did marginally affect protein levels in R6/2 mice. Gene expression was analysed using the ΔΔCt method and normalized to *Tbp* and *18* *S*. Samples were processed in parallel on gels/blots, and stain-free method was used for normalization of protein levels. Full-length blots are presented in Supplementary Fig. 3. Data represent mean ± SEM of the following number of 8–11 animals/group. Statistical significance was determined by 2-way ANOVA with Bonferroni post hoc test for multiple comparisons. *p < 0.05, **p < 0.01, ***p < 0.001, ****p < 0.0001

Altered gene expression in skeletal muscle has been shown to reflect HD progression^8^, including activation of apoptotic and NFκ-B pathway transcripts in R6/2 mice^26^. We therefore evaluated gene expression changes related to muscle damage and cachexia in gastrocnemius skeletal muscle, and the effects of 2 and 4 weeks of ghrelin treatment on these alterations. We found an increase in apoptotic and NFκ-B pathway transcripts (increased expression of *Caspase 3*, *Caspase 8*, *Smad3* and *Traf-5*) and an increase in *Creb1*, which is activated during muscle injury^27^, in R6/2 compared with WT mice. Notably, 4 weeks of ghrelin treatment normalized skeletal muscle mRNA expression of *Caspase 8* (*p* = 0.008), *Traf-5* (*p* < 0.0001), and *Creb1* (*p* < 0.0001). Expression of *Caspase 3* was not significantly altered with ghrelin administration (*p* = 0.08) (Fig. 2a). In addition, we evaluated gene expression related to mitochondrial biogenesis function previously shown to be altered in HD^28^. We found an increased expression of *Sirt1* (p = 0.011), which is activated during energy deficits in skeletal muscle (reviewed in ref.^29^), and a decreased expression of *Mfn2* (p = 0.022), which is required for mitochondrial fusion^30^, in R6/2 compared to WT mice skeletal muscle (Fig. 2b). Additionally, a trend towards decreased mRNA expression of *Nrf2* (Fig. 2b), an oxidative stress response gene^31^, was found in R6/2 skeletal muscle compared to WT mice. There was however, no significant effect on mitochondrial gene expression after 4 weeks of ghrelin administration.

**Figure 3:**
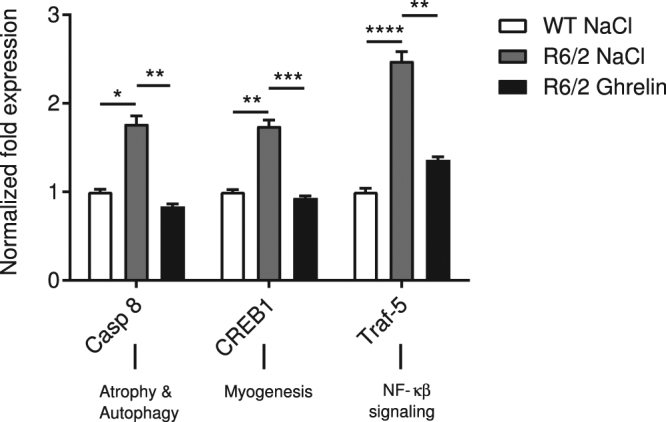
Ghrelin administration (for 2 weeks) normalizes altered gene expression profile in R6/2 mouse skeletal muscle. Normalized skeletal muscle (gastrocnemius) gene expression at 12 weeks and after 2 weeks of ghrelin administration. Expression of *Casp8*, *Creb1* and *Traf-5* were significantly increased in R6/2 compared to WT mice, and after 2 weeks of ghrelin administration normalization of *Casp8* and *Creb1 expression*, and in part *Traf-5* expression could be seen. Data represent mean ± SEM of the following number of 8–11 animals/group. Statistical significance was determined by 2-way ANOVA with Bonferroni post hoc test for multiple comparisons. *p < 0.05, **p < 0.01, ***p < 0.001, ****p < 0.0001.

At the protein level, a trend towards increased TRAF-5 was seen in R6/2 mice skeletal muscle compared to wildtype littermates (p = 0.07) (Fig. 2c). This increase was not seen in R6/2 mice compared with wildtype mice following 4 weeks of ghrelin administration (p > 0.99). Activated CREB was increased in vehicle-treated R6/2 mice at the protein level compared with wildtype littermates, which was not detected following ghrelin administration (p > 0.99) (Fig. 2d). [TRAF-5 protein: genotype p = 0.0005, F(1, 28) = 15.6, treatment p = 0.437, F(1, 28) = 0.6219, interaction p = 0.8687, F(1, 28) = 0.02782; pCREB/CREB: genotype p = 0.6249, F(1, 36) = 0.2431, treatment p = 0.0816, F(1, 36) = 3.209, interaction p = 0.5726, F(1, 36) = 0.3243]. To investigate whether altered skeletal muscle gene expression could be rescued at an earlier time point, *Caspase 8*, *Creb1* and *Traf-5* expression was assessed after 2 weeks of ghrelin administration. Increased expression of *Caspase 8*, *Creb1* and *Traf-5* was evident in R6/2 mice compared with WT mice at 12 weeks of age (Fig. 3). Interestingly, 2 weeks of ghrelin administration normalized mRNA expression of *Casp8* (*p* = 0.003) and *Creb1* (*p* = 0.0009), and *Traf-5* (*p* = 0.0014) mRNA expression was partly normalized. The mRNA expression level of *Acta1*, a muscle contractility component, remained unaltered after ghrelin administration (Fig. 2a).

**Figure 4:**
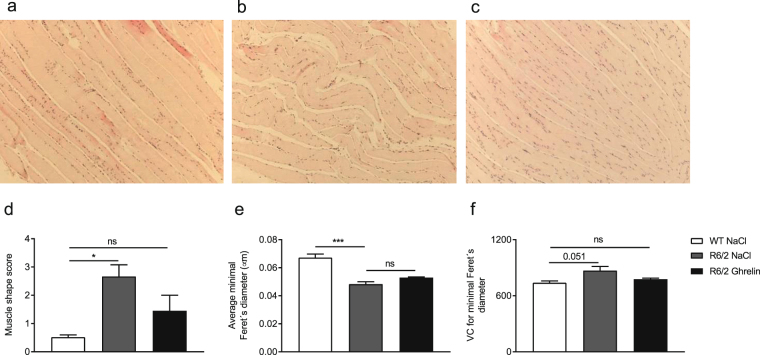
Ghrelin administration normalizes skeletal muscle morphology in R6/2 mice. Skeletal muscle (femoris) morphology was evaluated in R6/2 mice after ghrelin or vehicle administration for 4 weeks and compared with WT littermates. Muscle tissues were stained with hematoxylin and eosin and images were captured under bright-field conditions with a 10x objective. The scale bar at the bottom right in each panel represents 50 μm. Representative images illustrating longitudinal skeletal muscle from WT treated with vehicle (**a**), R6/2 treated with vehicle (**b**), and R6/2 treated with ghrelin (**c**). The skeletal muscle morphology of vehicle treated R6/2 mice had a convoluted shape and non-muscular infiltrations were found, instead of the parallel aligned fibers seen in vehicle treated wildtype littermates. An improvement was seen after 4 weeks of ghrelin treatment in R6/2 mice, and the morphology was comparable to wildtype littermates. A morphology score (**d**) was given to longitudinal skeletal muscle (femoris) indicating presence of convoluted shape, muscle fibre degeneration and grade of non-muscular infiltrations. We found a significant increase in score in vehicle treated R6/2 mice compared to WT mice, normalized following ghrelin administration. Minimal Feret’s diameter (**e**) was measured of cross sectional skeletal muscle (femoris) and the variation coefficient was calculated (**f**) in R6/2 mice and WT littermates after 4 weeks of treatment with either ghrelin or vehicle. There was a significant decrease in diameter and a trend towards higher diameter variability in R6/2 mice compared to WT mice after 4 weeks of vehicle treatment, but no improvement was seen following ghrelin treatment. Although skeletal muscle diameter and variability was not improved by ghrelin administration, the variability in ghrelin treated R6/2 mice showed no significant difference compared to WT littermates. Data represent mean ± SEM n = 3 animals/ group. Statistical significance was determined by One-way ANOVA: *p < 0.05, ***p < 0.001

Additionally, we investigated whether gene and protein expression changes were accompanied by skeletal muscle morphological alterations in hematoxylin-eosin stained femoris skeletal muscle. In vehicle-treated R6/2 mouse skeletal muscle, signs of atrophy were present morphologically, illustrated by fibres with convoluted shape and signs of non-muscular infiltration (Fig. 4b). Ghrelin administration normalized R6/2 skeletal muscle morphology (Fig. 4c). A morphological score, from 0 to 4, was given to assess morphological changes in shape and grade of non-muscular tissue infiltration. A significantly higher score in R6/2 (vehicle) compared to WT mice was found. However, no significant difference in R6/2 (ghrelin) compared to WT mice was seen (Fig. 4d). Minimal Feret’s diameter, as well as muscle diameter variability in cross-sectional fibres, has previously been shown by Briguet and colleagues to be reliable histological parameters to determine muscle degeneration in mouse models of dystrophy^32^. The variant coefficients, calculated using minimal Feret’s diameter, illustrate the variability of the fibre diameter^32^. Although Minimal Feret’s diameter was not affected by ghrelin administration, R6/2 mice displayed a decreased diameter in comparison to WT littermates (*p* = 0.0008) (Fig. 4e) where there was a beneficial effect on the variant coefficient. There was a strong trend towards an increase in variant coefficient when comparing R6/2 (vehicle) mice with WT littermates (*p* = 0.051) (Fig. 4f). Notably, in ghrelin administered R6/2 mice, there was no difference in the variant coefficient compared to WT mice (*p* > 0.99). Representative cross-sectional images are illustrated in Supplementary Fig. 4.

### Ghrelin administration does not affect glucose homeostasis in R6/2 mice

**Figure 5:**
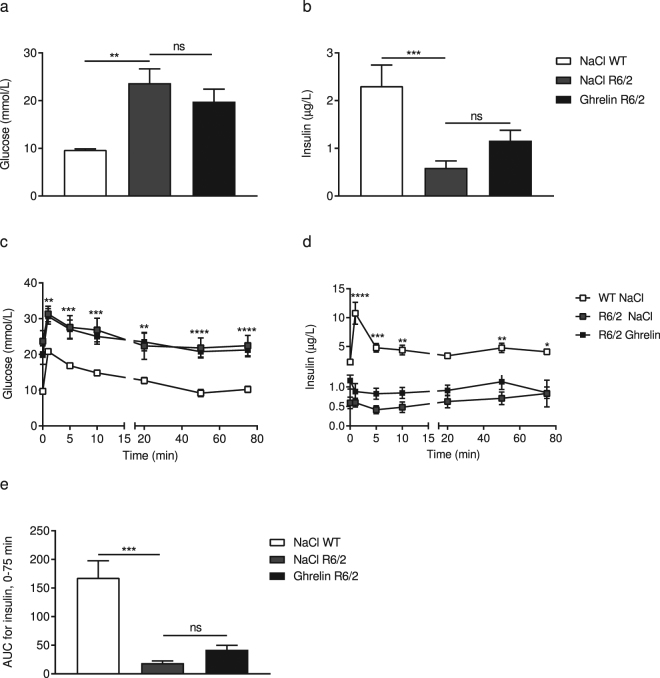
Effects of ghrelin administration on serum glucose homeostasis and insulin levels in R6/2 mice. Serum levels of glucose and insulin were measured at different time points (0, 90 s, 5, 10, 20, 50 and 75 min). A significant increase in basal glucose levels (**a**) and a significant decrease in basal insulin levels (**b**) were observed in 14-weeks old R6/2 mice compared with WT. Levels of glucose over time were increased in R6/2 mice (**c**), while insulin secretion was decreased (**d**). Ghrelin administration had a beneficial effect on insulin secretion in R6/2 mice (**e**), however the effect did not reach significance. Data represent mean ± SEM, WT vehicle, n = 9; R6/2 vehicle, n = 9; R6/2 ghrelin, n = 10. Statistical significance was determined by 2-way ANOVA with Bonferroni post hoc test for multiple comparisons. *p *<* 0.05, **p < 0.01, ***p < 0.001, ****p < 0.0001.

We have previously demonstrated reduced plasma levels of insulin and accompanying hyperglycaemia in R6/2 mice^33^. Therefore, we evaluated the effect of ghrelin on glucose homeostasis and insulin response in R6/2 mice and WT littermates. Mice were subjected to an intravenous glucose tolerance test (IVGTT) at the end of the study, at 14 weeks of age. In agreement with our previous studies^33^, basal serum glucose concentration was 2.4-fold higher and basal insulin concentration in serum was 3.9-fold lower in R6/2 mice (Fig. 5a and b). Moreover, glucose disposal was delayed during the IVGTT in these mice, while insulin response was decreased (Fig. 5c–e). No significant effect on basal glucose and insulin levels were observed in ghrelin treated R6/2 mice (Fig. 5). Basal levels of glucose and insulin were also measured after 2 weeks of treatment (halfway through the study) (Table 1). At this point, R6/2 mice had 2.3–fold higher glucose levels and 3-fold lower insulin levels; no significant effect of ghrelin treatment was evident.

**Table 1:** Effects of ghrelin on glucose and insulin levels after 2 weeks of administration.

	WT vh	R6/2 vh	R6/2 ghrelin	*p*-value^#^	*p*-value^§^
**Glucose metabolism**					
Basal glucose (mmol/L)	7.85 ± 0.55	18.21 ± 1.99	17.88 ± 2.03	0.002	>0.9999
Basal Insulin (μg/L)	1.18 ± 0.23	0.40 ± 0.08	0.43 ± 0.07	0.038	>0.9999
HOMA-IR	11.17 ± 2.43	8.68 ± 2.04	7.72 ± 1.47	>0,9999	>0,9999
HOMA-β	147.3 ± 28.41	13.66 ± 2.86	19.47 ± 5.99	<0,0001	>0,9999

The table shows the effects of ghrelin after 2 weeks of treatment. Data are expressed as mean ± SEM. Two-way ANOVA with Bonferroni post hoc test for multiple comparisons was used to analyse data. ^#^Represents the *p*-value between WT and R6/2 treated with vehicle, and ^§^represents the *p*-value between vehicle and ghrelin treatment in R6/2 mice. A *p*-value below 0.05 was considered significant.

HOMA-IR and HOMA-β were calculated after 2 and 4 weeks of treatment (Tables 1 and 2). HOMA-β in R6/2 mice was significantly decreased compared to wildtype littermates, while no change was seen in HOMA-IR (Tables 1 and 2

**Table 2:** Effects of ghrelin on body composition, and levels of glucose and insulin.

	WT vh	R6/2 vh	R6/2 ghrelin	*p*-value^#^	*p*-value^§^
**Body composition**					
Fat mass (g)	7.5 ± 0.55	4.79 ± 0.64	6.36 ± 0.54	0.01	0.26
Lean mass (g)	24.77 ± 0.31	20.80 ± 0.49	21.63 ± 0.36	<0.0001	0.97
Fat mass (%)	21.87 ± 1.63	18.38 ± 2.22	22.45 ± 1.29	0.76	0.42
Lean mass (%)	78.12 ± 1.63	81.60 ± 2.21	77.57 ± 1.29	0.76	0.43
**Glucose metabolism**					
Basal glucose (mmol/L)	9.70 ± 0.19	23.69 ± 2.98	19.81 ± 2.60	0.0002	>0.9999
Basal Insulin (μg/L)	2.31 ± 0.44	0.59 ± 0.15	1.16 ± 0.22	0.0007	0.28
AUC Insulin, 0–75 min	168 ± 29.57	18.85 ± 3.84	42.21 ± 7.79	0.0009	> 0.9999
HOMA-IR	24.57 ± 4.70	14.19 ± 2.85	27.86 ± 8.35	>0,9999	0,5157
HOMA-β	187.8 ± 36.79	21.61 ± 9.54	43.3 ± 9.35	0,0002	>0,9999

The table shows the effects of ghrelin after 4 weeks of treatment. Data are expressed as mean ± SEM. Two-way ANOVA with Bonferroni post hoc test for multiple comparisons was used to analyse data. ^#^Represents the *p*-value between WT and R6/2 treated with vehicle, and ^§^represents the *p*-value between vehicle and ghrelin treatment in R6/2 mice. A *p*-value below 0.05 was considered significant.

). [Basal glucose levels: genotype p < 0,0001, F(1, 35) = 36.08; treatment p = 0.3933, F(1, 35) = 0.7469; interaction p = 0.2744, F(1, 35) = 1.233; Basal insulin levels: genotype p = 0.0002, F(1, 35) = 16.78; treatment p = 0.9212, F(1, 35) = 0.009924; interaction p = 0.0661, F(1, 35) = 3.598; Glucose levels: time points p < 0.0001, F(6, 245) = 18.31, Genotype + treatment p < 0.0001, F(3, 245) = 84.63, interaction p = 0.9999, F(18, 245) = 0.2012; Insulin levels: time points p < 0.0001, F(6, 240) = 13, genotype + treatment p < 0.0001, F(3, 240) = 78.58, interaction p < 0.0001, F(18, 240) = 5.598; AUC for insulin: genotype p < 0.0001, F(1, 35) = 67.76, treatment p = 0.0283, F(1, 35) = 5.234, interaction p = 0.2444, F(1, 35) = 1.402].

### Ghrelin administration does not affect inflammatory markers in R6/2 mouse serum

Inflammatory markers were assessed in order to evaluate a possible overt effect of ghrelin administration. In addition, ghrelin administration has been suggested to affect the immune system^34^ and previous studies have shown circulating levels of immune markers to be altered in R6/2 mice^35,36^. At the end of the study, at 14 weeks of age and 4 weeks of ghrelin administration, we did not here observe an altered cytokine profile in R6/2 mice compared with WT mice treated with vehicle (Supplementary Table 2) and ghrelin administration did not elicit an immune response. The R6/2 mice used in this study are not at end stage of disease, which might explain why the subtle R6/2 immune response is not detected in serum.

### Effects of ghrelin administration on fatty acid metabolism in liver and white adipose tissue

**Figure 6:**
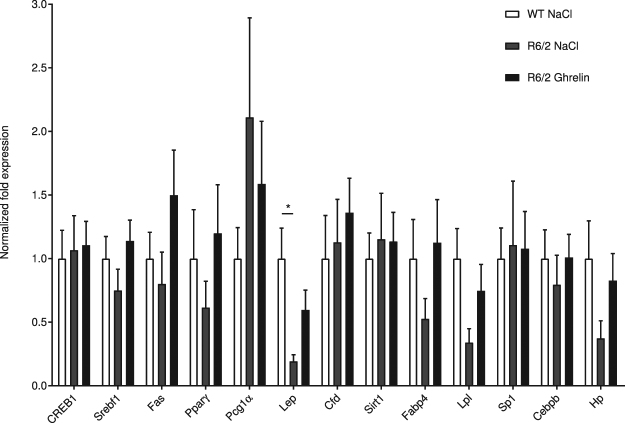
Gene expression changes in white adipose tissue after ghrelin administration. Normalized gene expression from intra-abdominal white adipose tissue of 14-week old saline treated R6/2 mice (R6/2 NaCl; grey bars), ghrelin treated R6/2 mice (R6/2 Ghrelin; black bars) and saline treated wild type littermates (WT NaCl; white bars). Here, we found ghrelin treatment to have a tendency to normalize leptin mRNA levels in R6/2 mice. We also observed trends towards recovery of reduced R6/2 gene expression for *Pparγ*, *Fabp4*, *Lpl* and *Hp*, following ghrelin administration. Gene expression was analysed using the ΔΔCt method and normalized to *Gusb*, *Hsp90ab1* and *Tbp*. N = 8–11/group (n = 5 for the *Pparγ* R6/2 Ghrelin group). Data represent means ± SEM, *p < 0.05.

We next assessed whether ghrelin administration affects liver and adipose tissue mRNA levels of the key lipogenic enzyme fatty acid synthase (*Fas*) and its related transcription factors, sterol regulatory element binding protein-1 (*Srebf1*) and peroxisome proliferator-activated receptor-γ (*Pparγ*). The mRNA level of adipose tissue-derived hormone Leptin (*Lep*)^37^, which is a key factor in energy metabolism, was also assessed. In 14-week old R6/2, mouse intra-abdominal white adipose tissue, *Lep* mRNA levels were significantly decreased compared with WT littermates; this alteration was partly normalized by 4 weeks of ghrelin treatment (Fig. 6).

Fatty acid metabolites have been shown to be altered in HD^38^ and we therefore evaluated circulating levels of triglycerides (TG) and cholesterol. Levels of TG in 14-week old R6/2 mice were increased; administration of ghrelin only marginally affected serum TG levels in R6/2 mice (Supplementary Table 2). In contrast, hepatic TG levels were unaltered in 14-week old R6/2 mice and unaffected by ghrelin administration (Supplementary Table 2). Similarly, levels of total cholesterol and hepatic total cholesterol were also unaltered in 14-week old R6/2 mice (Supplementary Table 2).

### Ghrelin administration rescues lack of nest building behaviour in R6/2 mice

**Figure 7:**
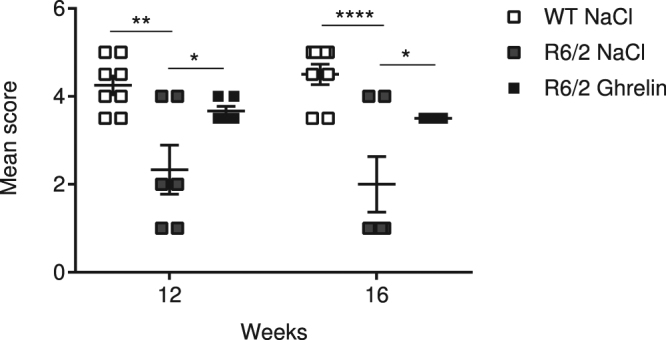
Ghrelin rescues lack of nest building behaviour in R6/2 mice. The nest building deficits present in R6/2 mice in comparison to wild-type mice was rescued by ghrelin administration. Ghrelin was here administrated once daily for 6 weeks starting at 10 weeks. Nesting material was introduced into mice home-cage (mice assessed in pairs), and morning after at 12 and 16 weeks, nest quality was scored as 1–5 as described in Deacon *et al*.^39^. Score 1 represents an almost untouched nestlet, for score 3 >50% was shredded but not into a specific nest site, and score 5 represents an almost perfect nest were >90% of the nestlet was used to build the nest as a crater and the walls were higher than the mouse body height. Data represent mean ± SEM, n = 6–8/group. Statistical significance was determined by 2-way ANOVA with Bonferroni post hoc test for multiple comparisons. *p < 0.05, **p < 0.01, ****p < 0.0001.

Nest building ability in R6/2 mice and their wildtype littermates was assessed after 2 and 6 weeks of ghrelin or vehicle administration in a separate cohort of mice. Nesting material was introduced into home-cages (mice were assessed in pairs), and the following morning, at 12 and 16 weeks (Fig. 7), nest quality was scored as previously^39^, according to untouched nesting material and the quality of the nest (for representative images of scores see Supplementary Fig. 6). We found that nest building deficits were evident in vehicle treated R6/2 mice after 2 and 6 weeks of treatment compared to WT littermates. Notably, ghrelin administration rescued nest building deficits seen in R6/2 mice. [genotype + treatment p = 0.0005, F(2, 17) = 12.13; time points p = 0.5770, F(1, 17) = 0.3235; interaction p = 0.2466, F(2, 17) = 1.522; subjects (matching) p = 0.0002, F(17, 17) = 6.581].

## Discussion

In HD, accumulating evidence supports the concept that pathology is not limited to the brain. Mutant huntingtin is expressed throughout the body and induces widespread effects. Studies in animals have shown that central pathology in HD is susceptible to modulation by treatments targeting peripheral tissues, supporting modulation of peripheral targets as therapeutic approaches in HD^3^. HD is associated with a catabolic state, resulting in weight loss and a lower body mass index in both HD mouse models^11^ and in human HD^40,41^. Importantly, a higher BMI is associated with a slower disease progression^42^ and a catabolic profile can be detected in early HD patients^41,43^, preceding the onset of overt symptoms. This suggests that alterations in energy homeostasis participate in early HD pathogenesis, opening up for the potential of normalizing energy metabolism with a beneficial effect on disease progression.

The R6/2 transgenic HD mouse model replicates many features of human HD, including weight loss, which has been shown to be linked to increased energy metabolism^11^. Here, we demonstrate that daily injections of ghrelin for 4 weeks postpone R6/2 mouse weight loss by one week. As previously demonstrated, ghrelin treatment results in body weight gain in wild-type mice^23,24^; in line with this, our chosen dosage strategy significantly increased body weight in wild-type mice (see Supplementary Fig. 1a). In both R6/2 and WT mice, body weight gain did not result in significant alterations in either lean or fat mass, suggesting that ghrelin exerts effects on both tissues, resulting in an accumulated effect on body weight.

Wasting of skeletal muscle is commonly observed in HD patients^6,7,44^. The underlying mechanisms of muscle wasting in HD are not known, but may be a direct consequence of the presence of mutant huntingtin in myocytes^45^. Mutant huntingtin forms inclusion bodies in muscle cells of both HD patients^46^ and mouse models^2,47^, and in skeletal muscle, there is a mutant huntingtin triggered gene expression phenotype present in both mouse models of HD and in HD patients^8,48^. HD myocytes have been shown to exhibit increased expression of genes encoding, for example, chaperones and heat shock proteins, and muscle-specific mRNAs have been shown to be altered^8,48^. The increased expression of genes involved in apoptosis and autophagy^25,26^ suggests that these mechanisms may contribute to a catabolic phenotype and muscle wasting. In line with this, we here confirmed that R6/2 mouse skeletal muscle exhibits a catabolic gene expression profile. We found that R6/2 mice exhibit increased skeletal muscle gene expression of Caspases 3 and 8, which are involved in the apoptotic pathway, confirming what has previously been shown^25^ and that cAMP response element binding protein (CREB) is increased, which has been shown to be activated in response to muscle damage^27^. Alterations in gene expression were accompanied by changes in protein expression. Interestingly, ghrelin administration reversed the catabolic gene expression profile already after 2 weeks of ghrelin treatment. Importantly, the normalization of gene expression was accompanied by normalized muscle morphology, which is in accordance with previous studies where ghrelin inhibited doxorubicin induced apoptosis in skeletal muscle^49^. Mitochondrial dysfunction has been suggested as a key player in skeletal muscle pathology in HD. Downregulation of *Pgc1-α* and downstream gene expression plays a critical role in inducing deficits in mitochondrial biogenesis and cellular respiration^28^. *Sirt1* has been postulated to act as a sensor of energy deprivation and as a regulator of metabolism homeostasis, by activating *Pgc1-α* (reviewed in^29^). Increased expression of *Sirt1* has been shown to have a negative effect on muscle differentiation both *in vitro* ^50^, and *in vivo* ^51^ resulting in a negative impact in muscle repair. The here shown alterations in muscle mitochondrial gene targets (*Sirt1 and Mfn2*) in R6/2 mice compared to WT littermates, could suggest an energy deprived phenotype with decreased regeneration ability. Although not significant, a trend of improvement after 4 weeks of ghrelin treatment was seen in *Sirt1* expression levels.

The minimal Feret’s diameter and variant coefficient are quantitative and reliable measurements of muscle diameter and variability of cross-sectional fibres frequently used to study degeneration in skeletal muscle^32^. In our study, we found that R6/2 mice have a decreased muscle diameter, which was not altered by ghrelin administration. A higher variability in the diameter was seen in R6/2 mice, suggesting muscle fibre degeneration. This variability was not observed following ghrelin administration, suggesting that ghrelin exerts a beneficial effect, not only on gene expression profile, but also on morphological changes.

Alongside muscle atrophy, an altered body composition, accompanied by dysfunctional adipocytes, has been described in several HD mouse models^12,52–54^, and expression of mutant huntingtin in adipocytes has been shown to result in altered adipocyte function, such as reduced triglyceride storage within the adipocytes^53^. A feature of the HD hyper catabolic state has been suggested to be an abnormal fatty acid metabolism. The disrupted cholesterol observed in HD has been shown to be linked to mutant huntingtin’s effect on specific action on sterol regulatory element binding proteins (SREBP)^38^. Adipose tissue regulates metabolism and glucose homeostasis through the release of adipokines, such as leptin^55,56^. Previous studies have shown that leptin levels are decreased in R6/2 mice^53^. In agreement, we found lower leptin mRNA levels in R6/2 adipose tissue, and a tendency for normalization after 4 weeks of ghrelin treatment. These results agree with the observed decreased fat mass, and the tendency of increased fat mass with ghrelin treatment. We also observed altered expression of adipocyte genes and genes involved in adipocyte differentiation and maturation in R6/2 mice, which were partly normalized by ghrelin administration. Potentially, ghrelin administration could play a beneficial role in HD adipose tissue via normalization of adipose tissue energy storage capacity.

Hyperglycaemia and reduced insulin levels have previously been demonstrated in the R6/2 mouse model^33^, which were confirmed in the present study. Furthermore, glucose disposal was delayed upon an IVGTT in these mice, while insulin response was decreased. A tendency for improvement was seen in basal glucose and insulin levels in R6/2 mice after 4 weeks of ghrelin treatment. In addition, we observed decreased HOMA-B in R6/2 mice, but no difference was seen in HOMA-IR. Studies in humans, rodents and *in vitro* have given contradictory results on glucose-induced insulin secretion followed by ghrelin administration. Although an inhibitory effect seems to be the most prominent, found in mice^57^, rats and in pancreatic β-cells^58^, beneficial effects on insulin secretion are also present by central and peripheral actions^59,60^. In this study, we did not find any significant effect on either serum insulin or glucose levels, or on the calculated HOMA-B or HOMA-IR in R6/2 mice.

Metabolic hormones play key roles, not only in whole body energy metabolism, but also in neurodegenerative processes and ghrelin has been shown to effect brain function, increasing neuronal survival^61^. Ghrelin analogues are already in clinical use, and have been shown to improve muscle atrophy^62^ as well as exert beneficial effects on cognition^21,63^. In HD, the motor phenotype is accompanied by a reduced ability to perform everyday tasks. Nest-building behaviour in mice requires organization of a complex set of behaviours, such as fine motoric skills, as well as cognitive function^64^. Nesting ability has been shown to be affected in both Alzheimer^65^ and Parkinson mouse models^66^. In the present study, ghrelin administration robustly improved R6/2 nest building capacity, suggesting a beneficial effect of ghrelin on behavioural deficits. Whether these effects on behaviour are related to the observed improved peripheral effects of ghrelin needs further investigation.

Taken together, our data demonstrates beneficial effects of ghrelin administration on body weight and skeletal muscle in R6/2 mice. In addition, effects, even if mild, on multiple tissues, all point towards normalization of energy metabolism aspects. Importantly, ghrelin also improves behavioural aspects. Our study supports the notion that affecting energy metabolism may exert a beneficial effect on HD disease progression. Current notion suggests that it is time to target the whole body in HD. We here provide data that opens up for new therapeutic avenues and further studies evaluating the underlying effects of ghrelin administration in HD are warranted.

## Materials and Methods

### Animals

All experimental procedures performed on mice were carried out in accordance with the approved guidelines in the ethical permit approved by The Malmö/Lund Animal Welfare and Ethics Committee (ethical permit number: M15-5).

Male transgenic R6/2 HD mice (expressing exon 1 of the HD gene)^22^ and their wild-type (WT) littermates (Jackson Laboratories, Bar Harbor, ME, USA), obtained by crossing heterozygous R6/2 males with C57BL/6 females, were used in this study. Tail tips were sent on dry ice to Laragen (Laragen Inc., CA, USA) for CAG repeat length determination, by polymerase chain reaction assay. The R6/2 mice used in this study had a CAG repeat size ranging from 266–328. The CAG repeat size of our R6/2 colony, results in a disease progression slower than that of the R6/2 mouse with 150 CAG repeats, as described by Morton and co-workers in 2009^67^. Mice were housed in groups with *ad libitum* access to chow food and water under standard conditions (12 h light/dark cycle, 22 °C). During the final 2 weeks of treatment, mice also had access to wet food on the cage floor.

Ghrelin (Rat, mouse; 100 µl, 150 μg/kg; Phoenix Pharmaceuticals, Belmont) or sterile NaCl (vehicle; 100 µL) was injected subcutaneously (s.c.) once daily for either 2, 4 or 6 weeks, from the age of 10 weeks onwards (prior to R6/2 weight loss). 150 μg/kg ghrelin has previously been shown to exert beneficial effects on body weight^23^. Body weight was monitored twice/week, commencing one week prior to ghrelin administration.

At the end of the 4 week ghrelin treatment, mice were subjected to an intravenous glucose tolerance test (IVGTT). *D*-glucose (1 mg/g) was injected into a tail vein of anesthetized mice ((midazolam 0.4 mg/mouse (Dormicum, Hoffman-La-Roche, Basel, Switzerland)) and a combination of fluanison (0.9 mg/mouse) and fentanyl (0.02 mg/mouse; Hypnorm, Janssen, Beerse, Belgium). Blood was obtained at different time points (0, 90 s, 5 min, 20 min, 50 min and 75 min) by retro-orbital bleeding. Serum obtained from blood samples was collected by centrifugation at 2000 x *g*, for 10 min, at 4 °C, and immediately frozen to −80 °C.

Following the IVGTT, body composition was measured using the Lunar Prodigy dual energy x-ray absorptiometry (DEXA; GE Lunar Corp., Madison, WI) and thereafter mice were euthanized and brain, liver, intra-abdominal adipose tissue, skeletal muscle and pancreas were dissected. Tissue samples were snap-frozen in liquid nitrogen and stored at −80 °C until further use.

### Serum analyses

Serum glucose levels were measured using the glucose oxidase method (Thermo Trace, Victoria, Australia), and levels of insulin were determined by radioimmunoassay (RIA; Linco Research Inc., St. Louis, MO, USA). The homeostatic model assessment (HOMA) was calculated to measure insulin resistance (IR) and pancreatic beta-cell function (β). HOMA-IR was calculated as follows: (Insulin X Glucose)/22.5, and HOMA-β as follows: (20 X Insulin)/(Glucose-3.5)^68^.

Cytokine levels (mouse pro-inflammatory 7-Plex kit measuring IFN-γ, IL-1β, IL-2, IL-4, IL-5, IL-6, IL-10, IL-12, TNF-α, mKC) were quantified using Meso Scale Discovery (MSD) assay, according to manufacturer’s protocol.

Serum triglyceride levels were measured using triglyceride quantification colorimetric kit (Biovision, Milpitas, CA, USA), according to manufacturer’s protocol. In all assays, samples were assayed in duplicates.

### Histology

Skeletal muscle femoris were dissected, placed into 4% paraformaldehyde in phosphate buffered saline (0.01 M) for two days and then sequentially moved into increasing concentrations of ethanol (50, 70, 90 and 100%). Sections (7 µm) were mounted on glass slides and stained according to Mayer’s Hematoxylin - Eosin staining protocol. Digital images of the stained sections were used to identify the morphological features of the muscle. A bright-light microscope (Olympus U-HSCBM, Olympus, Tokyo, Japan) with a 10x magnification objective, digital camera and image capture software (cellSens Dimensions 1.11 software; Olympus, Tokyo, Japan) were used.

### Quantification of muscle morphological changes

Morphology of longitudinal sections of skeletal muscle femoris from 14 week old mice was investigated and scored on the basis of presence of irregular shape and non-muscular infiltrations. Each section (15/mouse) was scored from 0 to 4, where sections with a score of 0 had no or small amount of non-muscular infiltrations, degeneration of muscle fibres, as well as no or small changes in morphological shape. Sections with a score of 4 had a severe infiltration of non-muscular tissue, muscle fibre degeneration and morphological changes in their shape and muscular arrangement, such as convoluted shape and gaps between the myofibres. The mean score ± SEM of each mouse (n = 3) was determined. The operator was unaware of the genotype of each sample during analyses and statistical analyses were performed independently.

### Quantification of muscle fibre diameter and diameter variability

Skeletal muscle femoris from wildtype mice administered with NaCl, and R6/2 mice with either ghrelin or NaCl (n = 3 in each group), were analysed. Minimal Feret’s diameter, which is the minimum distance of parallel tangents on opposite sides of the muscle fibre, was measured for all cross sectional fibres in each group using ImageJ 1.50 software (National Institutes of Health, Bethesda, MD). Muscle fibre diameter variability was analysed by calculating the variance coefficient of the minimal ‘Feret’s diameter as follows: variance coefficient = (standard deviation of the muscle fibre size/mean muscle fibre size) × 1000^32^.

### RNA extraction and cDNA synthesis

Total RNA was extracted from approximately 30 mg each of skeletal muscle gastrocnemius, liver tissue and approximately 50 mg adipose tissue, using the E.Z.N.A. Total RNA Kit II (Omega bio-tek, Norcross, Georgia, USA) before complementary DNA (cDNA) was synthesized using iScript cDNA Synthesis Kit (Bio-Rad Laboratories, CA, USA), according to manufacturer’s protocol. RNA concentration and purity were measured by a NanoDrop Lite spectrophotometer (Thermo Fisher Scientific, Wilmington, Delaware, USA).

### Real-time quantitative PCR

SsoAdvanced Universal SYBR Green Supermix from Bio-Rad Laboratories was used for RT-qPCR and performed following manufacturer’s protocol. All RT-qPCR plates were run on a CFX96 touch real-time PCR detection system (Bio-Rad, CA, USA). Primers utilized for RT-qPCR validations (see Supplementary Table 1) were designed using either QuantPrime^69^ or PrimerQuest from Integrated DNA Technologies (http://eu.idtdna.com/PrimerQuest). The efficiency of each primer pair was tested before use by performing a standard curve, and the efficiency criteria for using a primer pair was 90% < E < 110%, with an R^2^ cut-off >0.990. Housekeeping genes, TATA-binding protein (*Tbp*) and *18* *S*, were used for normalization of skeletal muscle tissue, and Glucuronidase beta, *Gusb*; Heat Shock Protein 90 kDa Alpha (Cytosolic), Class B Member 1, *Hsp90ab1*; and *Tbp* were used for normalization of adipose tissue RT-qPCR. Changes in gene expression were calculated using the CFX manager software program (Bio-Rad, CA, USA), using the ΔΔCt method with a fold change cut-off at ≥1.5 and *p* < 0.05 considered significant. All samples were run in triplicate and relevant positive and negative controls were run on each plate.

### Protein extraction

Proteins were extracted from approximately 30 mg of skeletal muscle gastrocnemius. The muscles were homogenized using a MP FastPrep-24 Tissue and Cell Homogenizer in 1 mL low salt lysis buffer containing 10 mM HEPES, 10 mM KCl, 1.5 mM MgCl2, 0.1 mM EDTA, 0.1 mM EGTA, 1 mM dithiothreitol (DTT), pH 7.9 supplemented with protease inhibitors (Complete, Roche, Basel, Switzerland) and phosphatase inhibitors (PhosSTOP, Roche, Basel, Switzerland). The homogenized muscle tissue was then sonicated for 3 × 5 s at 4 °C (Q500 Sonicator, QSonica, CT, USA). Homogenates were vortexed for 15 s, placed on ice for 10 min, vortexed again for 15 s and then centrifuged at 10,000 × g for 10 min at 4 °C to remove cell debris. The supernatant was collected and protein concentration was quantified using Pierce BCA Protein Assay Kit (Thermo Scientific, Rockford, IL, USA) according to manufacturer’s protocol.

### Western Blot

20 μg protein was mixed with 4× Laemmli Sample buffer complemented with 10% β-mercaptoethanol, before denaturing at 95 °C for 5 min. Lysates were run on Mini-PROTEAN TGX Stain-Free Precast Gels (Bio-Rad, CA, USA) and blotted on 0.2 μm PVDF membrane (BioRad CA, USA), which were blocked with 5% non-fat dry milk in Tris-buffered saline, pH 7.6, containing 0.1% Tween20 (TBS-T) for 1 h at room temperature (RT). Blocked membranes were then incubated with rabbit polyclonal TRAF-5 (H-257, Santa Cruz; 1:750), rabbit monoclonal phospho-CREB (S133, 87G3, Cell Signaling; 1:1000) and CREB (48H2, Cell Signaling; 1:1000) antibody in 5% non-fat dry milk in TBS-T overnight at 4 °C. After washing several times with TBS-T, membranes were incubated for 2 hrs with a secondary Goat anti-rabbit horseradish peroxidase-conjugated antibody (Dako; 1:3000). Signal was visualized using Western Blotting Luminol Reagent (Santa Cruz, TX, USA) and imaged using a ChemiDoc MP Imaging System (Bio-Rad, CA, USA). Stain-free technology was used as loading control and for protein normalization using Image Lab Software 5.2.1 (Bio-Rad, CA, USA).

### Nest building behavioural testing

Nest building is a spontaneous and complex behaviour of mice, requiring a high degree of organization, planning, problem solving, social ability^64^, as well as motoric skills for pulling, carrying, and bedding of nest material^70^. Nesting ability has been shown to be negatively affected in neurodegenerative diseases such as Alzheimer’s disease^65^ and Parkinson’s disease^66^. Testing the ability of nesting is an easy way of monitoring disease progression in neurodegenerative diseases, and may be a valuable way of testing the effect of treatments on cognitive and motoric dysfunction^65,66,71^. Nest building was assessed after 2 and 6 weeks (at 12 and 16 weeks of age respectively) of ghrelin administration in a separate cohort of mice. Mice of the same genotype and treatment were caged in duplicate overnight, with access to food and water, but with no environmental enrichment. Approximately 3 g of nesting material was placed in the cage, and untorn material was weighed the next morning. Nesting ability was assessed on a point scale as previously described^39^, from 1 to 5, analysing both untouched nesting material and shape of the nest. A score 1 was given when the nestlet was mainly untouched (>90% was intact) (Supplementary Fig. 6a), score 3 was given when the nestlet was mostly shredded but not until an identifiable nest site was seen (Supplementary Fig. 6b), and score 5 was given to an almost perfect nest, where >90% of the nestlet was torn up into a crater where the walls were higher than the body height (Supplementary Fig. 6c). Mice housed together were attributed the same score, and the mean score ± SEM of each group were determined.

### Statistical analyses

GraphPad Prism 7 was used to analyse all data (GraphPad Software Inc., San Diego, CA, USA). Results are presented as means ± SEM. One-way or two-way factor analysis of variance (ANOVA), with Bonferroni post-hoc test, were used for multiple comparisons.

F-values and degrees of freedom are presented for two-way ANOVA, giving the equality of means from each group. Differences with a *p* < 0.05 were considered statistically significant.

### Data availability

All data generated or analysed during this study are included in this published article, and its supplementary dataset.

## Electronic supplementary material

Supplementary Information
