# Supplementary material for: Ghrelin rescues skeletal muscle catabolic profile in the R6/2 mouse model of Huntington’s disease
Source: Sci Rep. 2017 Oct 24;7:13896. doi: 10.1038/s41598-017-13713-5 (PMC5654969; doi:10.1038/s41598-017-13713-5)
Supplement: Supplementary file 1 — Supplementary Information [file 41598_2017_13713_MOESM1_ESM.pdf]

Ghrelin rescues skeletal muscle catabolic profile in the R6/2 mouse model of Huntington´s disease

Marie Sjögren[1], Ana I. Duarte[1,2,3], Andrew C. McCourt[1], Liliya Shcherbina[4], Nils Wierup[4], Maria Björkqvist[1,*]

[1]Wallenberg Neuroscience Center, Department of Experimental Medical Sciences, Brain Disease Biomarker Unit, Lund University, Lund, Sweden.

[2]CNC - Center for Neuroscience and Cell Biology, Rua Larga, Faculty of Medicine (Pólo 1, 1st Floor), University of Coimbra, 3004-517 Coimbra, Portugal.

[3]Institute for Interdisciplinary Research (IIIUC), University of Coimbra, Casa Costa Alemão - Pólo II, Rua D. Francisco de Lemos, 3030-789 Coimbra, Portugal.

[4]Lund University Diabetes Centre, Neuroendocrine Cell Biology, Department of Clinical Sciences in Malmö, Clinical research center, Lund University, Malmö, Sweden.

Supplementary figures and tables

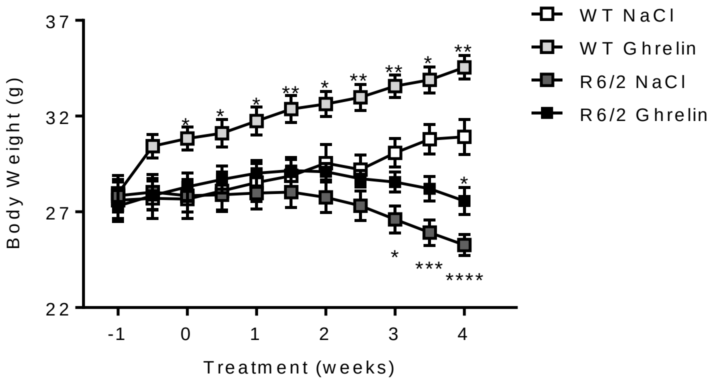

Supplementary Figure 1. Effects of ghrelin administration on body weight. Body weight was measured twice weekly, starting one week before ghrelin or vehicle injections. A significant increase in body weight was observed in 14-week old ghrelin treated WT mice compared to NaCl treated. Data represent mean ± SEM of the following number of animals/group: WT vehicle, n=9; R6/2 vehicle, n=9; R6/2 ghrelin, n=11. Statistical significance was determined by 2-way ANOVA with Bonferroni post hoc test for multiple comparisons. *p<0.05, **p<0.01, ***p<0.001, ****p<0.0001.

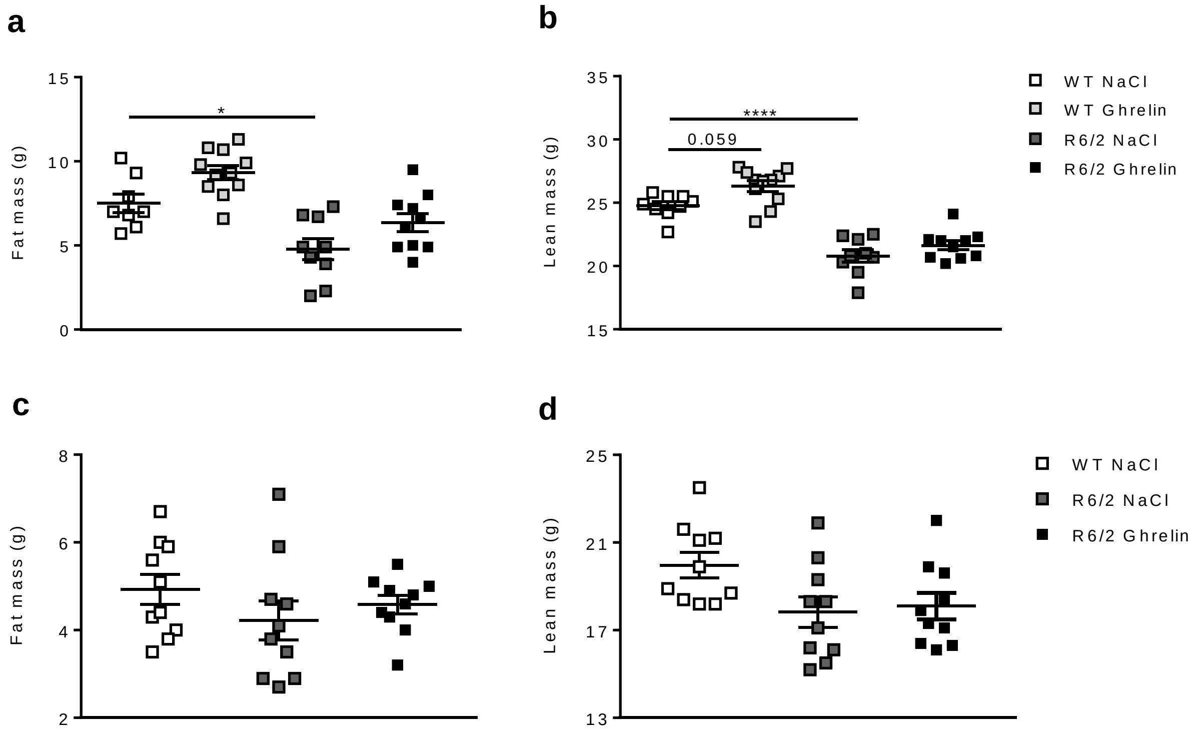

Supplementary Figure 2. Effects of ghrelin administration on body composition. Body composition was measured in R6/2 mice and WT littermates after 2 or 4 weeks of ghrelin or vehicle administration, using DexaScan. a. Fat mass (4 weeks). b. Lean mass (4 weeks). c. Fat mass (2 weeks). d. Lean mass (2 weeks). There was a significant decrease in both fat and lean mass in R6/2 mice compared to WT mice after 4 weeks of treatment. Data represent mean ± SEM of the following number of animals/group: WT vehicle, n=9; WT ghrelin, n=11; R6/2 vehicle, n=9; R6/2 ghrelin; n=10. Statistical significance was determined by 2-way ANOVA with Bonferroni post hoc test for multiple comparisons in the 4 weeks treated groups (a, b) and One-way ANOVA for the 2 weeks treated groups (c, d). *$p<0.05$, ****$p<0.0001$.

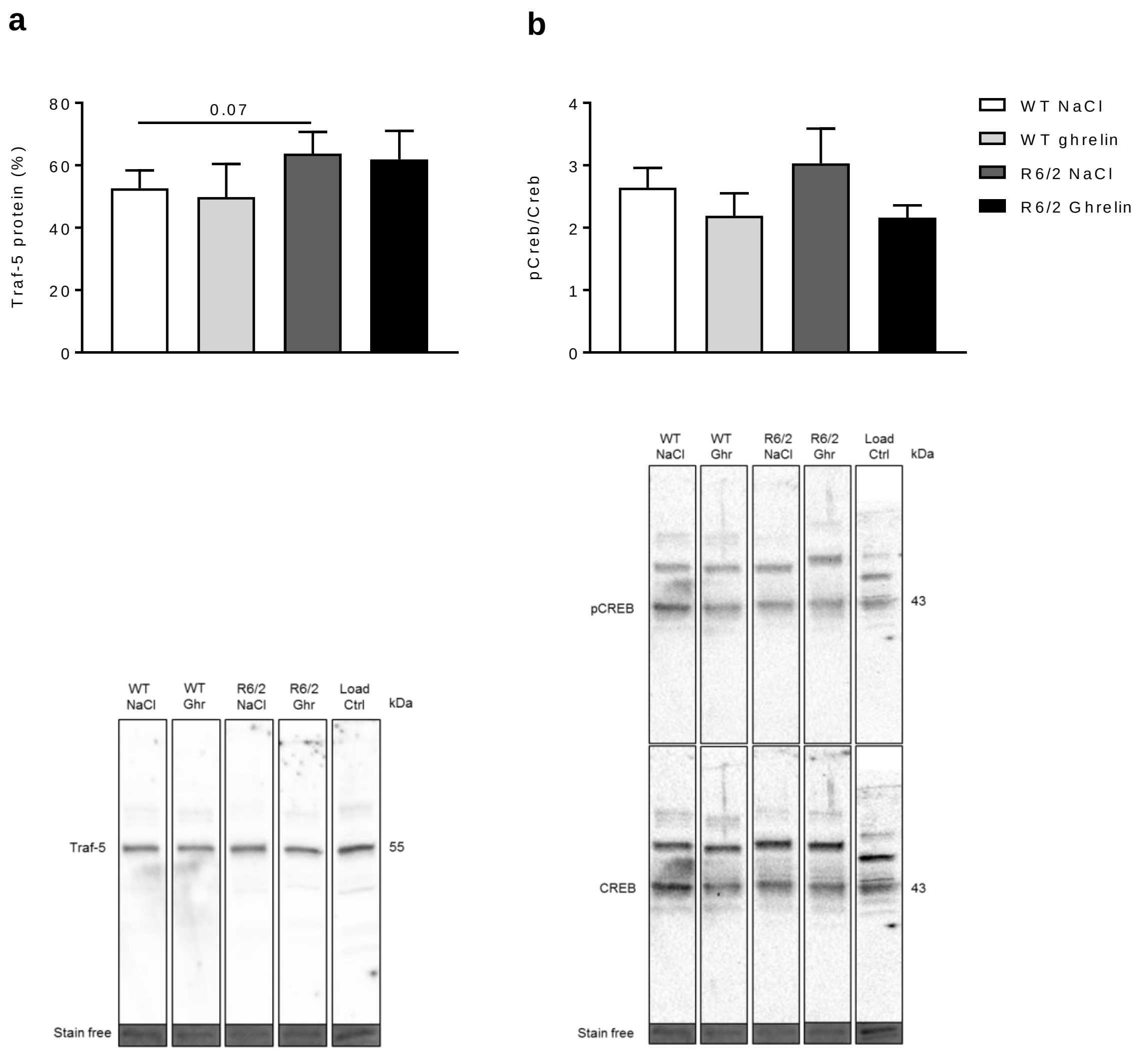

Supplementary Figure 3. Effect of ghrelin administration on skeletal muscle protein expression. The figure shows representative Western blot and densitometry protein levels in skeletal muscle (gastrocnemius) of 14-week-old R6/2 mice relative to WT littermates, treated with either ghrelin or vehicle. We could see a trend towards an upregulation of TRAF-5 protein levels (a) and activated CREB levels (b) in vehicle treated R6/2 mice compared to WT littermates. Ghrelin treatment showed signs of normalization in R6/2 mice since there was no change compared to wildtype littermates. Stain-free method was used for normalization. Data represent mean ± SEM of the following number of 8-11 animals/group. Statistical significance was determined by 2-way ANOVA with Bonferroni post hoc test for multiple comparisons.

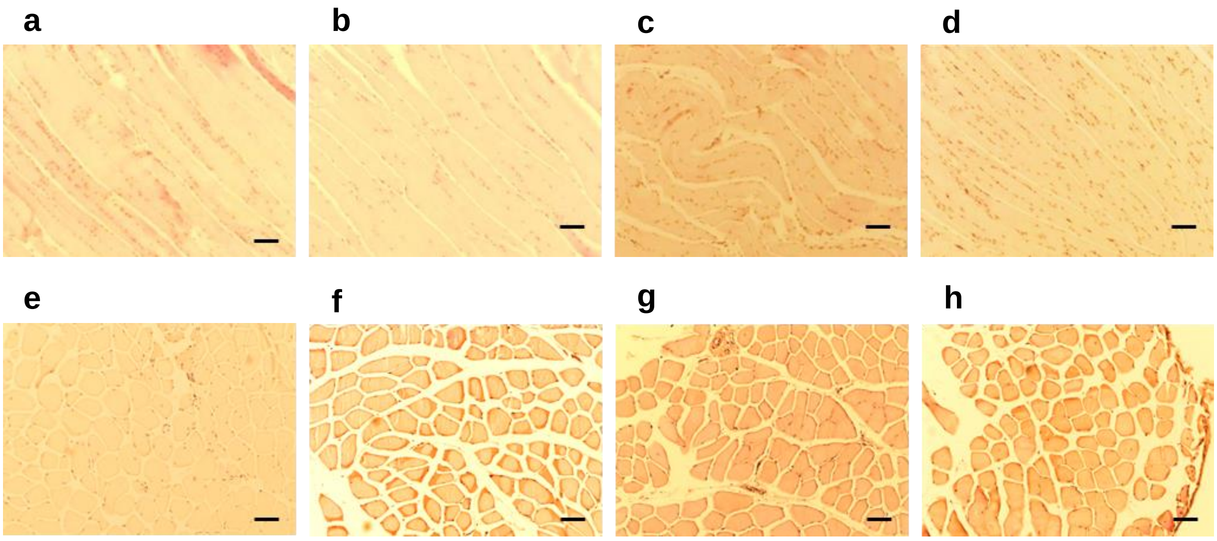

Supplementary Figure 4. Effects of ghrelin administration on skeletal muscle morphology. The morphology of the skeletal muscle (femoris) was studied in 14-weeks old R6/2 mice treated with either ghrelin or vehicle and compared with WT littermates. Muscle tissues were stained with hematoxylin and eosin and images were captured under bright-field conditions with a 10x objective. The scale bar at the bottom right in each panel represents 50 µm. Representative images illustrating longitudinal skeletal muscle from WT treated with vehicle (a), WT treated with ghrelin (b), R6/2 treated with vehicle (c), and R6/2 treated with ghrelin (d). Representative images illustrating cross-sectional skeletal muscle from WT treated with vehicle (e), WT treated with ghrelin (f), R6/2 treated with vehicle (g). d. R6/2 treated with ghrelin (h).

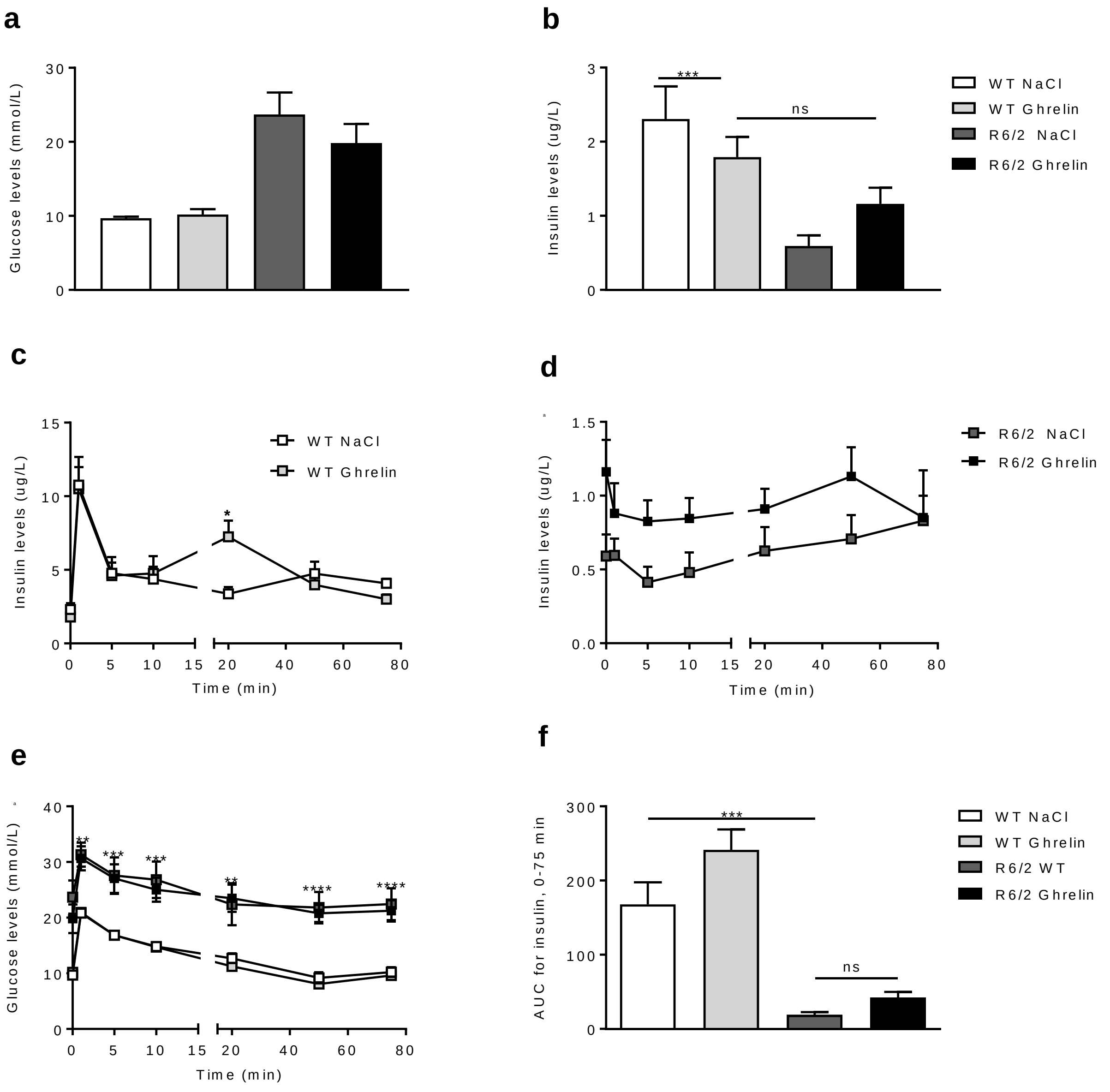

Supplementary Figure 5. Effects of ghrelin administration on serum glucose homeostasis and insulin levels in R6/2 mice. Serum levels of glucose and insulin were measured at different time points (0, 90s, 5, 10, 20, 50 and 75 min) a. Basal glucose levels. b. Basal insulin levels. c. Insulin levels over time in WT mice. d. Insulin levels over time in R6/2 mice. e. Glucose levels over time. f. AUC for insulin over time. A significant increase in basal glucose levels and a significant decrease in basal insulin levels were observed in14 weeks old R6/2 mice compared with WT. Levels of glucose over time were increased in R6/2 mice, while insulin secretion was decreased. Improvement in insulin secretion in WT mice was seen with ghrelin treatment 20 minutes after glucose injection. Data represent mean ± SEM of the following number of animals/group: WT vehicle, n=9; WT ghrelin, n=11; R6/2 vehicle, n=9; R6/2 ghrelin, n=10. Statistical significance was determined by 2-way ANOVA with Bonferroni post hoc test for multiple comparisons. *p<0.05, **p<0.01, ***p<0.001, ****p<0.0001.

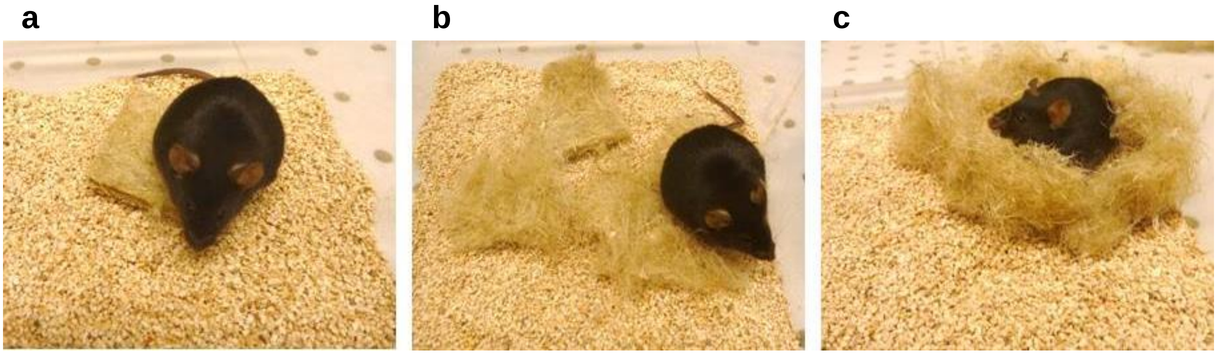

Supplementary Figure 6. Nesting scores. Figures represent different scores for the nesting behaviour testing. An almost untouched nestlet gives the score 1 (a), a score of 3 is given when more than 50% of the nestlet is shredded but no identified nest is found (b), and a score of 5 is given to an almost perfect nest where the nest walls are higher than the mouse body and have a form of a crater (c).

Supplementary Table 1. Primer sequences used for gene expression data.

Gene name	Forward Sequence (5' → 3')	Reverse Sequence (5' → 3')
18S	ACCGCAGCTAGGAATAATGGA	GCCTCAGTTCCGAAAACCA
Acta1	CACCATCGGCAATGAGCGTTTC	CCGCAGACTCCATACCGATAAAGG
Casp 3	TTGCCAGAAGATACCGGTGGAG	TCCAGGAATAGTAACCAGGTGCTG
Casp 8	AGTCACTTTGCCAGAGCCTGAG	TAGTTCACGCCAGTCAGGATGC
Cebpb	CGCGACAAGGCCAAGAT	GCTGCTCCACCTTCTTCTG
Cfd	CATGAACCGGACAACCTGCAATCT	ACGTAACCACACCTTCGACTGCAT
Creb1	GAGAGCTGGTATGTCAGGAATG	CCAGAAGAGATGCAGGAGAAAG
Fabp4	AGTGGGATGGAAAGTCGACCAC	CACGCCTTTCATAACACATTCCAC
Fas	AGTGCAAACCAGACTTCTACTGC	AAGGGTTCCATGTTCACACGAG
Gusb	CCGACTTCATGACGAACCAGTCAC	TGTCTCTGGCGAGTGAAGATCC
Hp	CGGAACGCCAACTTTAGATTTAC	CTTCTCGGGCACTGTACTATTC
Hsp90ab1	ATGATTAAACTAGGCCTGGGCATC	GCTTTAATCCACCTCTTCCATGCG
Lep	AGCAGCTGCAAGGTGCAAGAAG	AAAGCCACAGGAACCGACACAG
Lpl	CGCTCTCAGATGCCCTACAAAGTG	GTGTTGCTTGCCATCCTCAGTCC
Mfn2	ATGTGGCCCAACTCCAAGTGTC	TAACATCGATCCCAGGGCTGTC
Nrf2	AGATGACCATGAGTCGCTTGCC	AATCAGTCATGGCTGCCTCCAG
Pgc1α	CGTAAATCTGCGGGATGATGGAG	AGCGTCACAGGTGTAACGGTAG
Pparγ	AGGAAAGACAACGGACAAATCACC	ATTCGGATGGCCACCTCTTTGC
Sirt1	AGCAACATCTCATGATTGGCACCG	TCTGCCACAGCGTCATATCATCCA
Smad3	AATTACGGGCCATGGAGCTCTG	CACCAACACTGGAGGTAGAACTGG
Sp1	TGGGAAGCGCTTTACACGTTCG	GGCAGGCAAATTTCTTCTCTCCTG
Srebf1	GTGAGCCTGACAAGCAATCA	GGTGCCTACAGAGCAAGAGG
Tbp	TCTGAGAGCTCTGGAATTGTACCG	TGATGACTGCAGCAAATCGCTTG
Traf5	TTGTGGAGCAGCTGGAAGAACG	TCGGCACCGAGTTCAATTCTCTC
Ucp2	GGGTTCATGCCTTCCTTTCT	GAGATTGGTAGGCAGCCATTAG

Table shows primer sequences used for the validation of gene expression data. Efficiency and R2 of a standard curve were verified. Efficiency criteria for using a primer pair was 90%<E<110% and the R2 cut off was >0.990, with efficiencies below or above being excluded from this study.

Supplementary Table 2. Effects of ghrelin on cytokine, triglyceride and cholesterol levels.

	WT vh	WT ghrelin	R6/2 vh	R6/2 ghrelin	p-value #	p-value §
**Cytokines (pg/mL)**						
IFN-γ	0.31 ± 0.05	0.31 ± 0.05	0.42 ± 0.15	0.84 ± 0.58	> 0.9999	> 0.9999
IL-1β	0.32 ± 0.03	0.34 ± 0.04	0.61 ± 0.26	0.46 ± 0.09	0.81	> 0.9999
IL-2	1.02 ± 0.22	0.69 ± 0.08	3,21 ± 2,07	1.17 ± 0.16	0.75	0.81
IL-4	0.14 ± 0.03	0.35 ± 0.24	0.18 ± 0.03	0.20 ± 0.03	> 0.9999	> 0.9999
IL-5	1.11 ± 0.19	0.67 ± 0.07	2.34 ± 0.31	1.92 ± 0.28	0.005	> 0.9999
IL-6	175 ± 26.86	108.4 ± 16.59	323.1 ± 79.54	225.5 ± 29.7	0.14	0.67
IL-10	24.77 ± 2.54	22.33 ± 2.23	27.34 ± 7.11	28.01 ± 3.69	> 0.9999	> 0.9999
IL-12p70	8.34 ± 1.5	6.85 ± 0.86	13.15 ± 5.42	8.64 ± 1.26	> 0.9999	> 0.9999
KC/GRO	42.98 ± 7.51	43.18 ± 5.39	163.30 ± 115	69.60 ± 13.88	0.78	> 0.9999
TNF-α	5.13 ± 0.42	4.48 ± 0.39	10.07 ± 2.39	10.02 ± 1.02	0.07	> 0.9999
**Triglycerides (nmol/µL)**						
In liver	2,78 ± 0,32	3,25 ± 0,25	2,33 ± 0,23	2,83±0,16	>0,9999	0,94
In serum	18,92 ± 1.91	18,03 ± 2.75	36,37 ± 4.92	28,75±3.17	0,005	0,72
**Cholesterol (µg/µL)**						
In liver	0,21 ± 0,009	0,20 ± 0.01	0.21 ± 0.009	0.23 ± 0.01	>0,9999	0,90
In serum	1.04 ± 0.07	1.01 ± 0.06	1.04 ± 0.08	0.97 ± 0.06	>0,9999	>0,9999

Data are expressed as mean ± SEM and analysed by 2-Way ANOVA with Bonferroni post hoc test for multiple comparisons. # represents the p-value between WT and R6/2 treated with vehicle, and § represents the p-value between vehicle and ghrelin treatment in R6/2 mice. A p-value below 0.05 was considered significant.

Supplementary Table 3. Effects of ghrelin on body weight and composition, and levels of glucose and insulin.

	**WT vh**	**WT ghrelin**	**R6/2 vh**	**R6/2 ghrelin**	***p*-value #**	***p*-value §**
Body Weight (g)	30.92 ± 2.02	34.55 ± 1.57	25.27 ± 1.32	27.57 ± 1.97	< 0.0001	0.20
**Body composition**						
Fat mass (g)	7.5 ± 0.55	9.34 ± 0.41	4.79 ± 0.64	6.36 ± 0.54	0.01	0.26
Lean mass (g)	24.77 ± 0.31	26.32 ± 0.42	20.80 ± 0.49	21.63 ± 0.36	< 0.0001	0.97
Fat mass (%)	21.87 ± 1.63	26.10 ± 0.89	18.38 ± 2.22	22.45 ± 1.29	0.76	0.42
Lean mass (%)	78.12 ± 1.63	73.89 ± 0.89	81.60 ± 2.21	77.57 ± 1.29	0.76	0.43
**Glucose metabolism**						
Basal glucose (mmol/L)	9.70 ± 0.19	10.18 ± 0.73	23.69 ± 2.98	19.81 ± 2.60	0.0002	> 0.9999
Basal Insulin (µg/L)	2.31 ± 0.44	1.79 ± 0.27	0.59 ± 0.15	1.16 ± 0.22	0.0007	0.28
AUC Insulin, 0-75 min	168 ± 29.57	241.4 ± 27.33	18.85 ± 3.84	42.21 ± 7.79	0.0009	> 0.9999
HOMA-IR	24.57 ± 4.70	20.63 ± 3.66	14.19 ± 2.85	27.86 ± 8.35	>0,9999	0,5157
HOMA-β	187.8 ± 36.79	142.8 ± 25.88	21.61 ± 9.54	43.3 ± 9.35	0,0002	>0,9999

The table shows the effects of ghrelin after 4 weeks of treatment. Data are expressed as means ±SEM. 2-way ANOVA with Bonferroni post hoc test for multiple comparisons was used to analyse data. # represents the *p*-value between WT and R6/2 treated with vehicle, and § represents the *p*-value between vehicle and ghrelin treatment in R6/2 mice. A *p*-value below 0.05 was considered significant.

Supplementary Table 4. Effects of ghrelin on levels of glucose and insulin at midpoint of the study.

	WT vh	WT ghrelin	R6/2 vh	R6/2 ghrelin	*p*-value #	*p*-value §
**Glucose metabolism**						
Basal glucose (mmol/L)	7.85 ± 0.55	9.83 ± 0.88	18.21 ± 1.99	17.88 ± 2.03	0.002	> 0.9999
Basal Insulin (µg/L)	1.18 ± 0.23	1.37 ± 0.26	0.40 ± 0.08	0.43 ± 0.07	0.038	> 0.9999
HOMA-IR	11.17 ± 2.43	15.39 ± 3.46	8.68 ± 2.04	7.72 ± 1.47	>0,9999	>0,9999
HOMA-β	147.3 ± 28.41	121.5 ± 22.03	13.66 ± 2.86	19.47 ± 5.99	<0,0001	>0,9999

The table shows the effects of ghrelin after 2 weeks of treatment. Data are expressed as means ± SEM. 2-way ANOVA with Bonferroni post hoc test for multiple comparisons was used to analyse data. # represents the *p*-value between WT and R6/2 treated with vehicle, and § represents the *p*-value between vehicle and ghrelin treatment in R6/2 mice. A *p*-value below 0.05 was considered significant.
